# Durability of Basalt/Hemp Hybrid Thermoplastic Composites

**DOI:** 10.3390/polym11040603

**Published:** 2019-04-02

**Authors:** Claudia Sergi, Jacopo Tirillò, Maria Carolina Seghini, Fabrizio Sarasini, Vincenzo Fiore, Tommaso Scalici

**Affiliations:** 1 Department of Chemical Engineering Materials Environment, Sapienza-Università di Roma and UdR INSTM, Via Eudossiana 18, 00184 Roma, Italy; jacopo.tirillo@uniroma1.it (J.T.); mariacarolina.seghini@uniroma1.it (M.C.S.); 2 Department of Engineering, University of Palermo, Viale delle Scienze, 90128 Palermo, Italy; vincenzo.fiore@unipa.it (V.F.); T.Scalici@qub.ac.uk (T.S.); 3 School of Mechanical and Aerospace Engineering—Queen’s University Belfast, Ashby Building, Stranmillis Road, Belfast, BT9 5AH, UK

**Keywords:** polymer-matrix composites, hybrid, environmental degradation, natural fibres, durability, basalt fibres, hemp fibres

## Abstract

The Achilles heel of thermoplastic natural fibre composites is their limited durability. The environmental degradation of the mechanical properties of hemp and hemp/basalt hybrid-reinforced high-density polyethylene (HDPE) composites has been investigated with a special focus on the effects of water ageing and accelerated ageing, including hygrothermal and UV radiation. Modification of the matrix was carried out using a maleic anhydride high-density polyethylene copolymer (MAPE) as a compatibilizer. Hybridization of hemp fibres with basalt fibres and the incorporation of MAPE were found to significantly decrease the water uptake (up to 75%) and increase the retention of mechanical properties after accelerated ageing. Secondary crystallization phenomena occurring in the composites, as confirmed by differential scanning calorimetry (DSC) analysis, were able to counteract the severe combined effects of hygrothermal stress and UV radiation, with the exception of hemp-fibre composites where permanent damage to the fibres occurred, with 2% and 20% reduction in tensile strength and modulus, respectively, for a 30 wt % hemp fibre-reinforced HDPE.

## 1. Introduction

Over the last years, an increasing interest in producing composite materials from bio-sourced, recycled materials and their combinations has been triggered by the relatively new concept of Circular Economy [1], which can be thought of as a new industrial economy, meant to be restorative or regenerative by design [2]. This new paradigm is usually linked and confused with sustainable development [2], and its successful implementation is envisaged to be the solution that will allow for a reduction of environmental pollution and the creation of a closed loop for the products’ lifecycle [3]. These concerns are particularly worrying for composite materials, which exhibit desirable properties through the combination of two or more individual components, but are inherently difficult to recycle and mostly disposed of in landfills at the end of their lives. The replacement of synthetic fibres, mainly glass fibres, with lignocellulosic ones has gained momentum and has become one of the most important components of the future generation of industrial practices [4]. Despite the well-known favourable attributes of cellulosic fibre-reinforced composites [5], which have granted them established applications in the building and automotive fields, these biocomposites do not show mechanical properties comparable with those of glass fibre composites and, most importantly, their susceptibility to moisture absorption can significantly reduce their durability. Indeed, large-scale industrial adoption of natural fibre composites for load-bearing applications seems to be hindered by a lack of confidence in their structural performance, due to the reduced resistance to hygrothermal loading. Previous studies have shown that moisture induces degradation of the mechanical properties of natural fibre composites to a greater extent than synthetic counterparts [6,7,8,9,10,11]. This behaviour is ascribed to the fact that water molecules can have physical and chemical effects, not only on the polymer matrices but also on the inherently hydrophilic fibres and fibre/matrix interface [12].

Therefore, increasing use of biocomposites, especially for outdoor applications, firmly depends on how the degradation mechanisms are understood, handled, and tackled. In this regard, a straightforward solution is the hybridization of natural fibres with synthetic ones, which feature superior ageing resistance [13,14]. This approach has been widely exploited in literature, mainly with glass fibres, and the resulting composites have shown a suitable balance of thermal stability, mechanical properties, resistance against water absorption, and cost [7,15,16,17,18,19]. In a recent study [20], the authors investigated composite materials based on the combination of two natural fibres, namely basalt and hemp, in a high-density polyethylene matrix (HDPE). The hybridization with basalt fibres, scarcely addressed in literature, proved to be successful in increasing the tensile properties of hemp fibre composites, the overall crystallinity, and Vicat softening temperature.

The positive mechanical features of this formulation led to the need to address durability issues, with a view to broadening the application fields of such composites. The main aim is to assess the suitability of natural basalt fibres to increase the environmental resistance of natural fibre composites, based on the standard combination of a commodity polymer (HDPE) and bast fibres (hemp). Most studies dealing with accelerated ageing of natural fibre composites have addressed only the influence of water ageing [21,22,23], whereas in this study, much more complex and severe conditions were investigated, trying to simulate in-service conditions as much as possible. In this regard, basalt/hemp hybrid composites were subjected to moisture absorption and accelerated ageing cycles of hygrothermal stress and UV radiation for up to 56 days. In particular, the kinetics of water absorption and the effect of accelerated ageing on the tensile properties of the composites with and without a coupling agent (maleic anhydride-grafted high-density polyethylene (MAPE)) were studied in detail for the first time on hemp/basalt–HDPE composites.

## 2. Materials and Methods 

### 2.1. Materials

Eraclene MP90 (Melt Flow Rate at 190 °C/2.16 kg of 7 g/10 min and density of 0.96 g/cm^3^), an HDPE injection moulding grade from Eni Polimeri Europa, was used as matrix, while chopped basalt fibres were supplied by Incotelogy GmbH (Pulheim, Germany), with an average diameter of 13 μm and a nominal length of 3.2 mm. The fibres were surface-modified with a commercial sizing compatible with thermoplastics, specifically polypropylene. A commercially available compound of HDPE loaded with 30 wt % of hemp fibres, supplied by AFT Plasturgie (Fontaine les Dijon, France), was used to manufacture hemp composites. This masterbatch was then diluted with neat HDPE to obtain additional formulations. In order to increase the fibre/matrix interfacial adhesion, the same composite formulations were also produced with a matrix modified by an addition of 5 wt % Polybond 3009, supplied by Addivant Corporation (Danbury, CT, USA), which is a standard maleic anhydride-modified high-density polyethylene (MAPE), with high (0.8–1.2%) maleic anhydride content (Melt Flow Rate at 190 °C/2.16 kg of 5 g/10 min and density of 0.95 g/cm^3^).

### 2.2. Compounding and Processing

All formulations produced and tested are listed in Table 1. Raw materials (pellets and fibres) were fed in a co-rotating twin-screw extruder (Thermo Scientific Process 11, Thermo Fisher Scientific, Waltham, MA, USA) and the tensile specimens were obtained by injection moulding (Haake MiniJet II Pro, Thermo Fisher Scientific). Prior to extrusion, the hemp fibre compound was dried at 115 °C for 2 h. Two different temperature profiles (8 zones) were used for basalt and hemp-based formulations, due to differences in viscosity of the blends and thermal stability of the fibres, namely 170–180–190–200–200–190–180–180 °C and 150–160–170–180–180–170–160–160 °C, respectively. Screw speed was set at 150 rpm. The mould during injection moulding was maintained at 40 °C, while the loading cylinder was heated at 220 °C for basalt composites and at 180 °C for hemp and hybrid formulations. The injection procedure included two steps: a first injection step at a pressure in the range of 350–550 bar, depending on the fibre amount, for 10 s and a post injection step at 60 bar for an additional 10 s.

### 2.3. Water Absorption Studies

The effect of water absorption on composites was investigated in accordance with ASTM D570 (West Conshohocken, PA, USA). Five tensile samples for each configuration were oven-dried at 105 °C for 24 h. The conditioned specimens were immersed in distilled water at a temperature of 23 ± 2 °C until saturation. The samples were taken out periodically and weighed to the nearest 0.1 mg within 1 min, after wiping off the water on the surface of the samples with a cloth. The moisture content (percent weight gain) was calculated with Equation (1):

(1) $$M\left(\%\right)=\frac{w_{t}-w_{0}}{w_{0}}\times 100$$

where *w_t_* and *w*_0_ are the wet (at time *t*) and the conditioned weight, respectively. To investigate the mechanism and extent of deterioration of properties after ageing (at saturation), tensile tests were performed on re-dried aged samples. Drying of the wet samples was carried out at 50 °C for 5 days in an air oven.

The Fickian diffusion coefficient (*D*) was determined from Equation (2), in the range where the values of percent weight gain are less than 60% of the equilibrium value (*M_m_*):

(2) $$D=\pi \cdot {\left(\frac{k\cdot h}{4M_{m}}\right)}^{2}$$

where *M_m_* is the maximum moisture content (at equilibrium), *h* is the thickness of the composites, and *k* is the initial slope of a plot of *M*(*t*) versus *t*^1/2^, as expressed by Equation (3) [24]:

(3) $$k=\frac{M_{2}-M_{1}}{\sqrt{t_{2}}-\sqrt{t_{1}}}$$

### 2.4. Artificial Ageing

Composites were exposed to accelerated ageing in a climatic chamber (ACS 1200, Angelantoni, Massa Martana, Italy), by developing ageing cycles of hygrothermal stress (i.e., Phase A) and UV radiation (i.e., Phase B, constant irradiance equal to 5 W m^−2^) (Table 2) [25,26]. To complete a single step of 14 days, Phase A and Phase B were sequentially repeated 24 times, for a duration of 7 days, and this alternation was further repeated twice. During ageing, samples were removed from the chamber and tensile tested after 14, 28, 42, and 56 days. The parameters used for the accelerated ageing are based on a previous study of the last 10 years’ weather forecast data of the city of Palermo (Italy) [27].

### 2.5. Mechanical Characterisation of Composites

Type 1BA samples (l_0_ = 30 mm) in accordance with UNI EN ISO 527-2 (Milano, Italy) were used for tensile tests, which were carried out in displacement control, using a crosshead speed of 10 mm/min on a Zwick/Roell Z010 (Ulm, Germany). The strain was measured with a contacting extensometer. The results reported in the work are the average of at least five tests for each material formulation.

### 2.6. Thermal Characterisation of Composites

The thermal behaviour of the different formulations was investigated by differential scanning calorimetry (DSC) on three samples for each material type. Specimens were analysed in a Pyris I (Perkin Elmer, Waltham, MA, USA), according to the following thermal program: Heating from −25 °C to 180 °C (5 min hold), cooling to −25 °C, and heating to 180 °C, all steps at 10 °C/min. The measurements were carried out in nitrogen flow. The degree of crystallinity (*X_c_*) of the samples was calculated according to Equation (4):

(4) $$X_{c}\left(\%\right)=\frac{\Delta H_{m}}{\Delta H_{m}^{0}\left(1-w_{f}\right)}\cdot 100$$

where *ΔH_m_* represents the experimental enthalpy of melting of the sample (J/g), *ΔH_m_^0^* the enthalpy of melting for 100% crystalline HDPE (J/g), taken as 293 J/g [28], and *w_f_* is the weight fraction of fibres.

### 2.7. Morphological Characterisation

Composite fracture surfaces were investigated by scanning electron microscopy (SEM), using a Philips XL40 and a FE-SEM Zeiss Auriga (Oberkochen, Germany). All specimens were sputter coated with gold prior to examination.

## 3. Results and Discussion

### 3.1. Water Uptake

**Figure 1:**
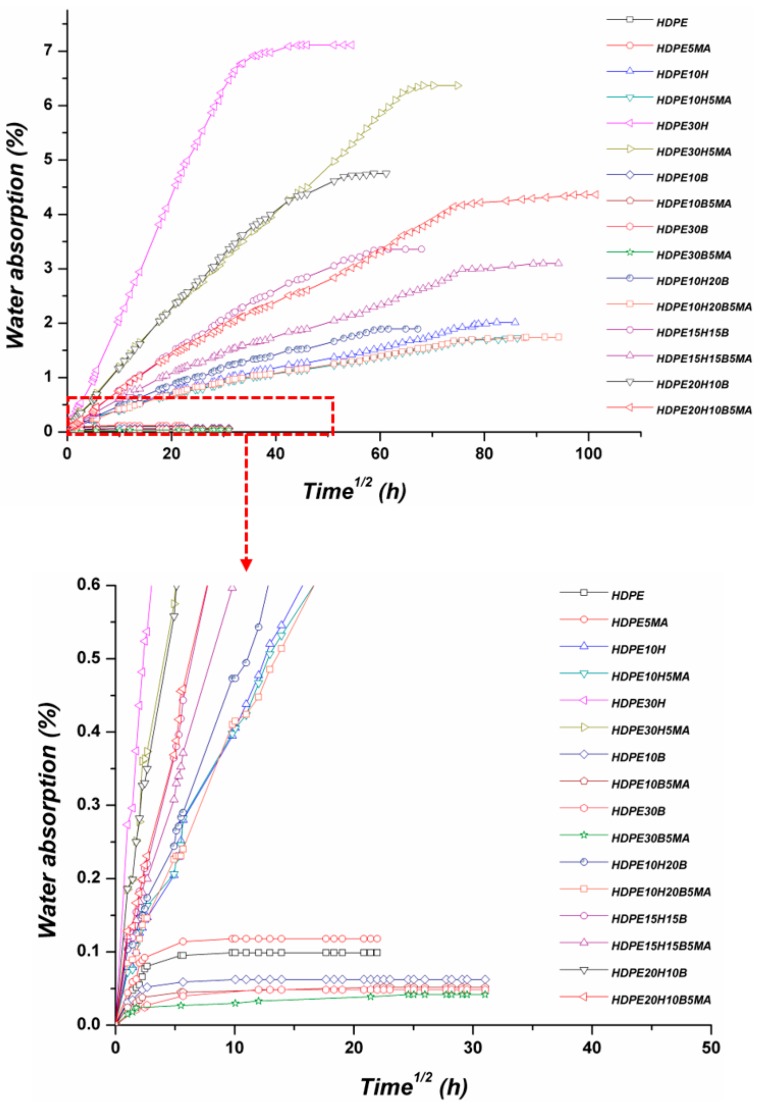
Water absorption curves of the different composites at room temperature.

Figure 1 shows water uptake evolution at room temperature for unmodified and 5 wt % Polybond 3009-modified composites as a function of the square root of immersion time. Each point represents the average of five samples. All the formulations exhibited a similar pattern of water uptake, in accordance with that reported in other studies [8,11,29]. After an initial stage with a linear and rapid increase, the water uptake attained a plateau without further increase in water absorption, thus exhibiting a Fickian mode of diffusion [18].

The equilibrium moisture content at saturation is summarized in Table 3 for the different formulations. Neat HDPE absorbed a very limited amount of water due to its hydrophobic nature, a value that was slightly enhanced by the addition of the coupling agent and its polar groups. Basalt and hemp fibres showed opposite effects on the water uptake of the resulting composites. From the data in Table 3, it is evident that with increasing basalt fibre content, a further decrease in the equilibrium moisture content was obtained compared to the neat matrix and this effect was even enhanced when the matrix was modified with MAPE, which can be ascribed to a sounder interfacial adhesion [20]. On the other hand, hemp fibres caused a significant increase in water uptake, which is only due to the hydrophilic character of lignocellulosic fibres, in particular to the presence of hemicellulose [10].

In fact, as previously noted, the amount of water absorbed by the neat matrix can be neglected, therefore the moisture absorption in natural fibre composites is a fibre-dominated property [16,30]. Natural fibres are indeed good examples of permeable fibres, which absorb moisture to a much larger extent than the matrix itself. The incorporation of the coupling agent in hemp-based formulations was effective in reducing the amount of water absorbed. In composite materials the transport of water can occur by three different mechanisms: (i) by diffusion through the matrix, (ii) by diffusion aided by the presence of defects in the matrix (voids, microcracks), and (iii) by capillarity along the fibre/matrix interface [10,21]. It is well known that the interfacial adhesion between natural fibres and hydrophobic polymers is far from being perfect, therefore it represents a preferential path for water ingress, thus easily exposing the hydrophilic groups of the fibre to the attack of water molecules. The resulting intermolecular hydrogen bonding further reduces the fibre/matrix interfacial adhesion, and the swelling of cellulose induces stresses at the interface that result in additional matrix microcracking, thus promoting water transport by capillarity.

For a matrix modified with MAPE, the occurrence of a better interfacial adhesion has already been demonstrated [20], and this improved adhesion limits water accumulation and prevents water from attacking hemp fibres. The effect of MAPE is dependent on its amount in the composite and its functionality (i.e., amount of grafted maleic anhydride) [31], as these two parameters control the effectiveness of stress transfer. In the present work, a relatively high amount of coupling agent (5 wt %), along with a high maleic anhydride content (0.8–1.2%), reduces the vulnerability of fibre hydroxyl groups to water attack, mainly through the covalent bonding of the grafted moiety to the hydroxyl groups on the hemp/basalt fibre surface, thus leading to a global decrease in water uptake.

The water absorption of hybrid composites was found to be favourably affected by the incorporation of basalt fibres, which is ascribed to the replacement of hydrophilic natural fibres with the basalt fibres in the resulting composites.

### 3.2. Kinetics of Water Absorption

The hypothesis of a Fickian mechanism was adopted for modelling the water absorption in composite materials and for determining the diffusion coefficient, *D*, with a 1D-approach (Equation (2)) [24,32,33,34]. Three different types of diffusion can occur: (i) Case I or Fickian diffusion, (ii) case II (and super case II), and (iii) case III (non-Fickian or anomalous diffusion) [35]. These three mechanisms can be conveniently distinguished, on a theoretical basis, by considering the particular shape of the absorption curve, which can be modelled by Equation (5) [35]:

(5) $$\frac{M_{t}}{M_{m}}=kt^{n}$$

where *M_t_* is the moisture content at time *t* (Equation (1)), *M_m_* is the moisture content at saturation, and *k* and *n* are constants. Parameter *n* is related to the mode of diffusion and assumes different values, depending on the particular case: For Fickian diffusion (case I), *n* = 0.5, while for case II, *n* = 1 (and for super case II, *n* > 1), and for case III (anomalous diffusion), 0.5 < *n* < 1. The values of *n* and *k* can be determined from the slope and the intercept of *M_t_/M_m_* vs. *t* in a logarithmic plot obtained from the experimental data, according to Equation (6):

(6) $$log\left(\frac{M_{t}}{M_{m}}\right)=log\left(k\right)+nlog\left(t\right)$$

Table 3 summarizes the values of the parameter *n*, resulting from the fitting for all the samples. Values of *n* for hemp fibre composites and hybrid composites are close to each other and point toward the value of 0.5, thus suggesting a Fickian diffusion mechanism in the composites. The deviation from the theoretical value can be ascribed to the occurrence of other mechanisms in natural fibre composites, such as fibre swelling, fibre/matrix interface weakening, matrix micro-cracking, and leaching [36,37].

Neat HDPE and basalt-based composites exhibited much lower values of *n*, where the rate of diffusion was likely to be slower than the polymer segmental mobility. This means that the equilibrium in the polymer, which is very limited in water amount, is quickly attained and maintained. The values of the diffusion coefficient are listed in Table 3, which appear to be in agreement with those reported by other researchers, i.e., in the range 10^−11^–10^−13^ m^2^/s [11,32,35,38,39]. The increase in basalt fibre content resulted in lower values of the diffusion coefficient, while the hemp fibres showed an opposite influence. This is due to the hydrophilic nature of natural fibres that made the water molecules ingress easier in the resulting composite materials.

It is interesting to note that the magnitude of the diffusion coefficient for hemp-based composites was lower than that for basalt-based composites. In a previous publication [20], it was demonstrated that both fibres have a nucleating effect in the HDPE matrix, but hemp fibres appeared to outperform the basalt ones. The higher crystallinity of the resulting composites could have hindered the diffusion of water molecules during the initial stages. Inclusion of basalt fibres decreased the diffusion coefficient in hybrid composites, and furthermore, in this case, the decrease in diffusion coefficient appears to be related to the relative increase in crystallinity [20]. The incorporation of MAPE reduced the diffusion coefficient even further, due to the improved fibre/matrix adhesion [20] that, by reducing the gaps in the interfacial region and by limiting the amount of available hydrophilic groups on the hemp fibres, hindered the diffusional process.

### 3.3. Effect of Water Absorption on Tensile Properties of Composites

**Figure 2:**
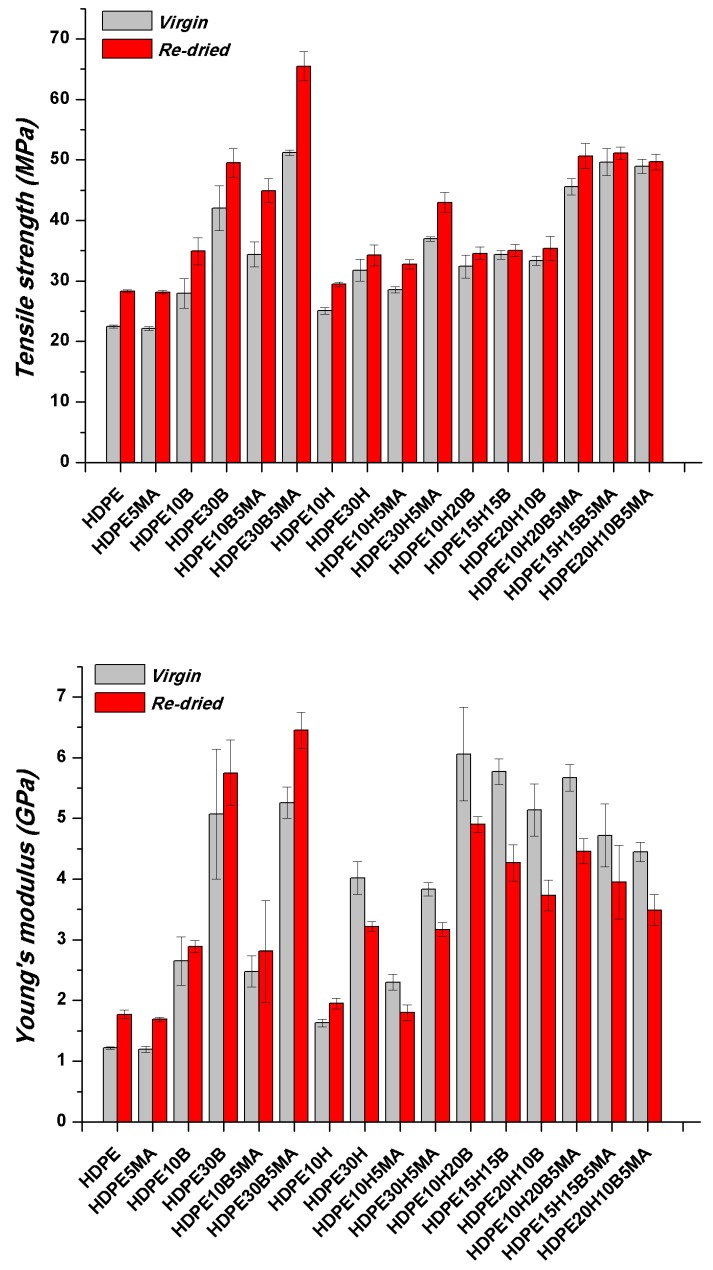
Tensile strength and Young’s modulus of high-density polyethylene (HDPE) and hemp/basalt fibre hybrid composites before and after water ageing.

Tensile tests were carried out on re-dried samples after the saturation level (up to a maximum of 6026 h) was achieved (re-dried) and compared with the values of pristine specimens, in order to investigate the effect of water ageing on the mechanical properties (Figure 2). Contrary to previous reports, where the prolonged exposure to moisture resulted in a significant strength and modulus degradation [11,16,18,29,35], in the present work, ageing in water did not lead to a reduction of strength of the composite samples, though it was found to decrease stiffness of hemp fibre-reinforced composites. The reduction in mechanical properties is usually ascribed to damages located in the fibres and at the fibre/matrix interface. Natural fibres are known to swell after exposure to moisture and this can result in a loss of stiffness and in the development of shear stresses at the interface, which induce debonding of the fibres from the matrix.

**Figure 3:**
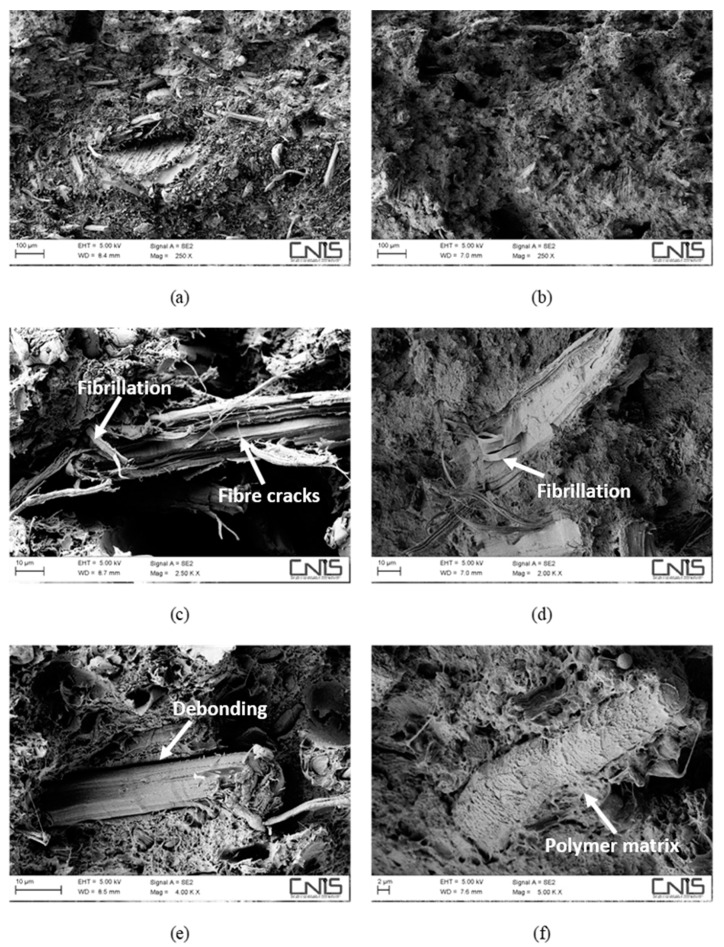
SEM micrographs detailing the fracture surface of water-aged composites, reinforced with 30 wt % of hemp fibres without (**a**,**c**,**e**) and with (**b**,**d**,**f**) a coupling agent.

Morphological investigation of the fracture surfaces by SEM supports this statement. In Figure 3, the morphology of hemp-based composites with and without a coupling agent is shown.

**Figure 4:**
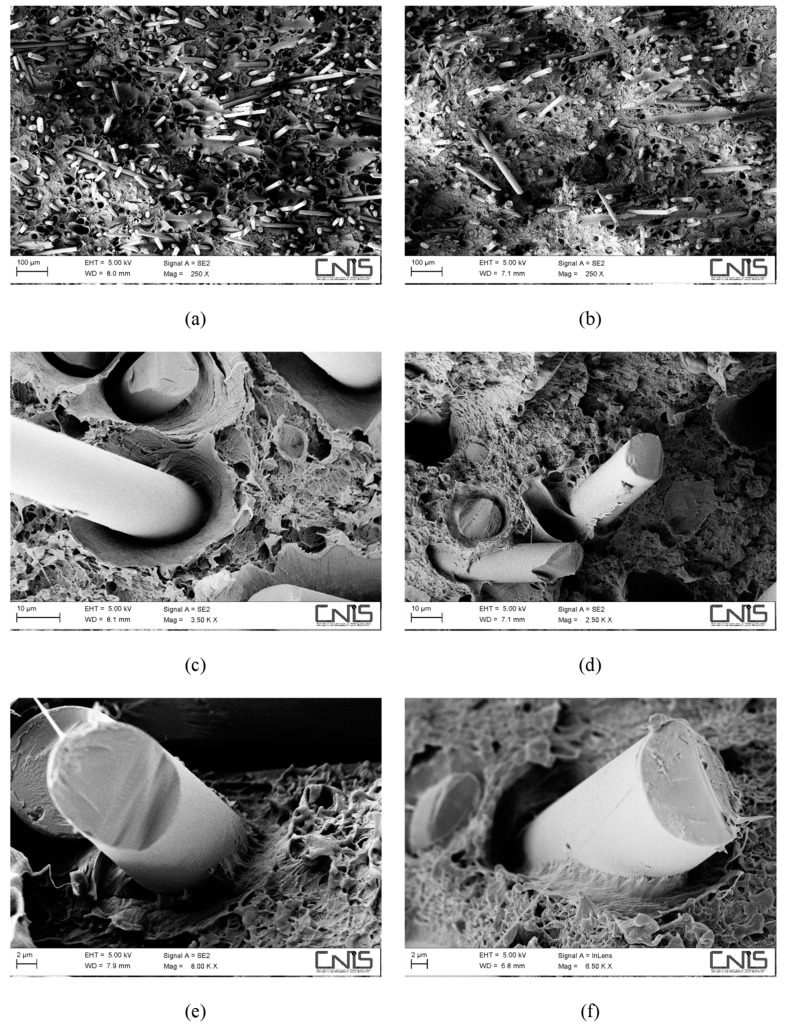
SEM micrographs detailing the fracture surface of water-aged composites, reinforced with 30 wt % of basalt fibres without (**a**,**c**,**e**) and with (**b**,**d**,**f**) a coupling agent.

The fibre/matrix interface, despite still being better when MAPE is present, appears to have been severely weakened. Comparing Figure 3e,f, wide gaps can be detected at the fibre/matrix interface in the non-compatibilized system (Figure 3e) without the presence of the polymer matrix sticking on the fibre surface, which instead can still be seen on compatibilized hemp fibres (Figure 3f). After water ageing, the most extensive damage was experienced by hemp fibres in the form of fibrillation (Figure 3c,d) and microcracks (Figure 3c), phenomena that can explain the reduction in the tensile modulus of the resulting composites, which is always reported to be reduced to a greater extent than the tensile strength [18,29]. For basalt-based composites, the same considerations apply (Figure 4), even if no reduction in tensile modulus was observed, mostly because basalt fibres are insensitive to moisture attack, at least at room temperature. Composites with a coupling agent exhibited a higher level of adhesion (Figure 4d,f) compared to untreated composites (Figure 4c,e), where longer fibre pull-outs as well as well-defined gaps between fibre and matrix were detected (Figure 4c), with fibres only barely wetted by the matrix. The matrix, in non-compatibilized systems, appears to be severely deformed around the fibres with limited adhesion.

For glass fibres, stress corrosion was reported, but it is unlikely to occur at room temperature [40]. The fact that both tensile strength and modulus (at least for basalt-based formulations) were found to increase with ageing is contrary to what is usually reported. It is worth noting that the same behaviour was found for neat HDPE and, therefore, it has to be ascribed to the polymer itself. A possible explanation, which will be discussed in the section on thermal properties, is related to the development of a secondary crystallization, where segments in the disordered regions of the polymer become mobile enough (due to the long ageing treatment and the plasticizing effect of water molecules) to rearrange themselves into lower-energy, more ordered structures, thus resulting in a progressive increase in the average crystallinity of the sample. This effect was able to balance the decrease in interfacial adhesion at least in the composites without natural fibres, which experienced severe damage. Hybrid composites were able to retain their properties to a larger extent than hemp fibre composites, thus suggesting a beneficial effect played by the incorporation of basalt fibres.

### 3.4. Accelerated Ageing

**Figure 5:**
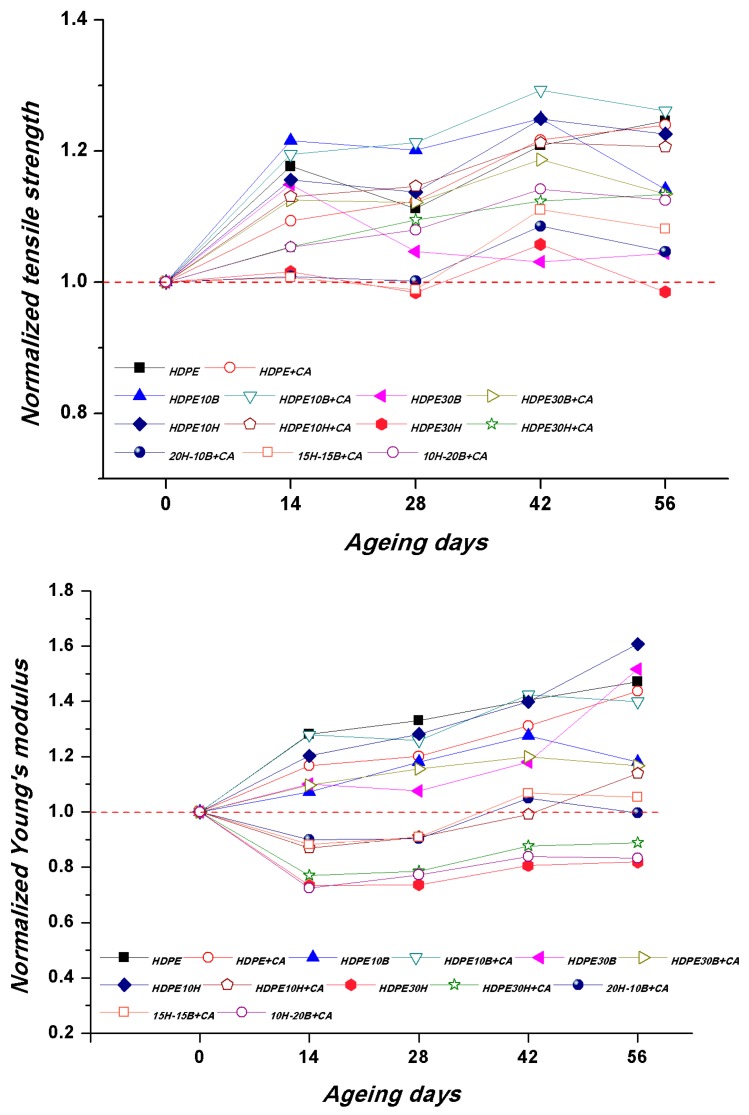
Evolution of the normalized mechanical properties as a function of exposure time.

Tensile tests were carried out on samples aged at different times, namely 14, 28, 42, and 56 days, in order to correlate the evolution of the mechanical properties with the exposure time (Figure 5).

Both tensile modulus and strength were found to improve with ageing time, and the effect was much more pronounced for strength compared to the modulus, which was found to decrease only for hemp fibre-reinforced composites. During accelerated ageing, several structural and chemical modifications can take place in HDPE polymeric chains, leading to a global degradation as a result of the combination of UV irradiation and moisture. These mechanisms are complex and can often occur simultaneously: chain-breaking due to homolytic and heterolytic dissociation with the appearance of methyl, isopropyl, or end unsaturation; branching and cross-linking phenomena, caused by radical addition, and oxidative phenomena, caused by either self-oxidation or photo-oxidation (mainly carbonyl groups) [41,42]. The changes are usually associated with an increase in density, hardness, and crystallinity [41], as well as in tensile strength and modulus [42]. The combined effects of the aforementioned chemical and structural modifications on the polymer crystallinity are very complex, as some changes can promote an increase in crystallinity, whereas others can lead to a crystallinity decrease. In the present case, the presence of fibres, which have exhibited a significant nucleation ability, also needs to be considered. DSC measurements were carried out in order to assess differences in crystallinity as a function of ageing time. Table 4 lists the melting and crystallisation parameters obtained from DSC curves, where the values of melting temperature (*T_m_*) and associated enthalpy (*ΔH_m_*) were calculated from the first heating scan, while the crystallisation temperature (*T_c_*) was calculated from the cooling curve.

For the sake of clarity, only the results belonging to the first and last ageing time have been included. From the results, a general increase in the degree of crystallinity was detected with increasing exposure time, coupled with a slight reduction of melting temperature, which suggests a decrease in the perfection of matrix crystallites [43,44], possibly related to the heterogeneous nucleation promoted by the presence of the fibres. It is speculated that during ageing, the material gets more packed due to modifications in the amorphous regions that are prone to oxidation with associated chain scission. These shorter molecules are more mobile so as to trigger secondary crystallization phenomena. Crystallization temperature did not exhibit a clear trend and differences among samples were limited to less than 2 °C. When comparing the crystallization temperatures with those of unaged specimens [20], a general decrease (in the range 6–8 °C) was detected after ageing. This suggests that the nucleating effect of basalt and hemp fibres, only partially counteracted in filled systems, the complex chemical changes occurring in the matrix, where the formation of bulkier groups (such as carbonyl) can increase the interchain distance, delay the onset of crystallization without affecting the overall amount of crystallinity [42].

**Figure 6:**
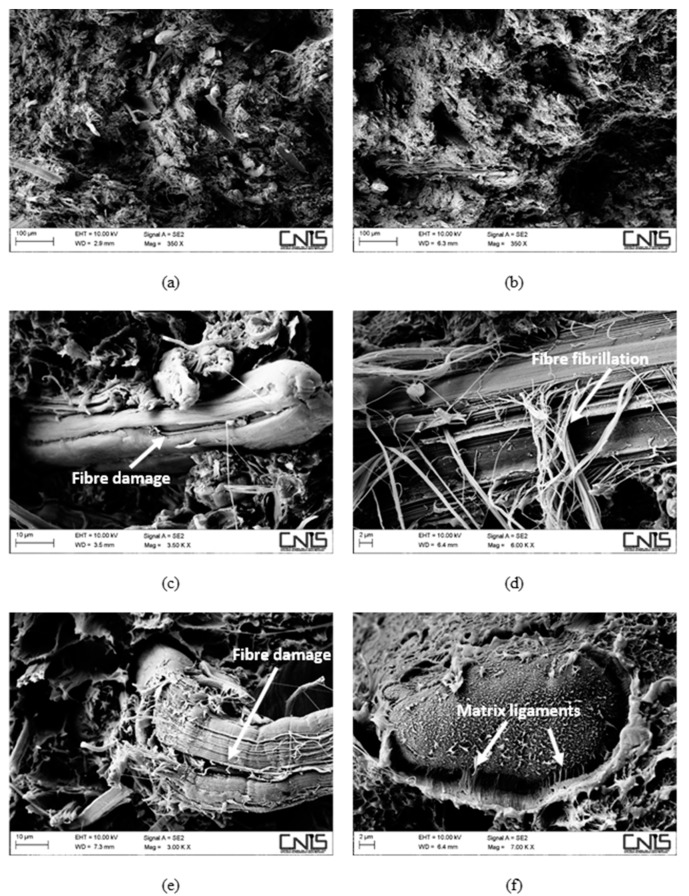
SEM micrographs detailing the fracture surface of aged composites, reinforced with 30 wt % of hemp fibres without (**a**,**c**,**e**) and with (**b**,**d**,**f**) a coupling agent.

**Figure 7:**
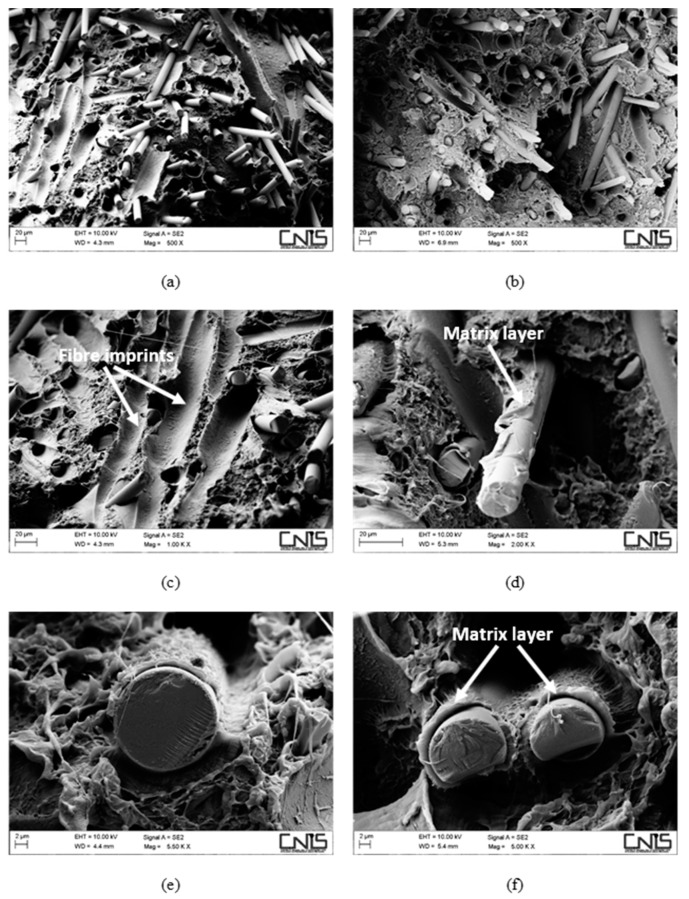
SEM micrographs detailing the fracture surface of aged composites, reinforced with 30 wt % of basalt fibres without (**a**,**c**,**e**) and with (**b**,**d**,**f**) a coupling agent.

An additional mechanism that is involved in degradation of the material is the damage located at the fibre/matrix interface and in the fibres. In this regard, it is worth noting the positive effect played by the presence of the coupling agent that limited the interfacial degradation, with matrix ligaments still adhering to the fibre surface (Figure 6f), while irreversible damage to hemp fibres in terms of fibre cracks (Figure 6c,d) and extensive fibrillation (Figure 6d) prevented the resulting composites from exploiting secondary crystallization effects to a large extent. On the contrary, hybrid composites benefited greatly from the incorporation of corrosion-resistant basalt fibres. In fact, basalt fibres were intact, while damage at the fibre/matrix interface was much more pronounced in non-compatibilized formulations, where extensive fibre imprints (Figure 7c) occurred more frequently compared to compatibilized systems and layers of the matrix were found to adhere to basalt fibres (Figure 7d,f), despite the severe ageing.

## 4. Conclusions

This study investigated prolonged moisture absorption and accelerated ageing of short hemp fibre and hemp/basalt hybrid-reinforced thermoplastic composites to assess their durability. Effects of basalt fibre hybridization, incorporation of MAPE on the water uptake, and the kinetics of moisture absorption of the hemp fibre composites were evaluated. The composites globally exhibited a Fickian mode of diffusion, but deviations were detected, which were ascribed to the dissolution of the lower molecular weight substances from the hemp fibres and damages to fibre/matrix adhesion. Ageing in water did not result in a reduction of the strength of the composite samples, while it was found to decrease the stiffness of hemp-based composites, due to their permanent damage. Tensile modulus and strength were found to improve with accelerated ageing time, and the effect was much more pronounced for strength compared to the modulus, which was found to decrease only for hemp fibre-reinforced composites. Secondary crystallization phenomena taking place in the composites were thought to be able to balance the deleterious combined effects of hygrothermal stress and UV radiation. In particular, basalt fibres proved to be effective in decreasing the water uptake (up to 75%) and increasing the retention of mechanical properties after accelerated ageing of hemp fibre composites, thus offering a suitable replacement of glass fibres after prolonged environmental exposure.

## Figures and Tables

**Table 1:** Formulation and description of composite samples produced and tested.

Designation of Samples	HDPE (wt %)	Basalt Fibre (wt %)	Hemp Fibre (wt %)	Coupling Agent (wt %)
NEAT HDPE	100	-	-	-
NEAT HDPE5MA	95	-	-	5
HDPE10B	90	10	-	-
HDPE30B	70	30	-	-
HDPE10B5MA	85	10	-	5
HDPE30B5MA	65	30	-	5
HDPE10H	90	-	10	-
HDPE30H	70	-	30	-
HDPE10H5MA	85	-	10	5
HDPE30H5MA	65	-	30	5
HDPE10H20B	70	20	10	-
HDPE15H15B	70	15	15	-
HDPE20H10B	70	10	20	-
HDPE10H20B5MA	65	20	10	5
HDPE15H15B5MA	65	15	15	5
HDPE20H10B5MA	65	10	20	5

**Table 2:** Values of the parameters of accelerated ageing.

	Theoretical Cycle (min)	T (°C)	RH (%)	Real Cycle (min)
**Phase A**				
Rain	75	20	95	75
Transition				8
Cold	40	2	50	32
Transition				8
Humid climate	115	35	87	107
Transition				6
Dry climate	70	50	56	64
Total	300			300
**Phase B**				
Humid climate + UV irradiation	120	35	87	120
Total A + B	420 (7 h)			420 (7 h)

**Table 3:** Summary of water absorption parameters for basalt/hemp composites.

Specimen ID	Equilibrium Moisture Content (%)	Diffusion Coefficient (m^2^/s)	*n* ^a^
NEAT HDPE	0.10 ± 0.03	1.41 × 10^−11^	0.21
NEAT HDPE5MA	0.12 ± 0.06	1.72 × 10^−11^	0.22
HDPE10B	0.06 ± 0.02	3.05 × 10^−11^	0.16
HDPE30B	0.05 ± 0.01	1.88 × 10^−12^	0.22
HDPE10B5MA	0.05 ± 0.03	2.66 × 10^−11^	0.12
HDPE30B5MA	0.04 ± 0.01	1.55 × 10^−12^	0.15
HDPE10H	2.01 ± 0.24	1.88 × 10^−13^	0.37
HDPE30H	7.11 ± 0.09	2.14 × 10^−13^	0.49
HDPE10H5MA	1.73 ± 0.14	1.81 × 10^−13^	0.35
HDPE30H5MA	6.36 ± 0.10	1.04 × 10^−13^	0.44
HDPE10H20B	1.89 ± 0.18	1.47 × 10^−13^	0.38
HDPE15H15B	3.35 ± 0.28	1.88 × 10^−13^	0.44
HDPE20H10B	4.75 ± 0.20	3.68 × 10^−13^	0.42
HDPE10H20B5MA	1.74 ± 0.02	1.06 × 10^−13^	0.35
HDPE15H15B5MA	3.09 ± 0.30	1.86 × 10^−13^	0.38
HDPE20H10B5MA	4.36 ± 0.42	2.88 × 10^−13^	0.42

^a^*n* is the exponent in Equation (5) and is related to the mode of diffusion.

**Table 4:** Thermal properties of composites obtained from differential scanning calorimetry (DSC) analysis.

Specimen ID	Ageing Time (14 days)				Ageing Time (56 days)			
	T_m_ (°C)	T_c_ (°C)	ΔH_m_ (J/g)	X_c_ (%)	T_m_ (°C)	T_c_ (°C)	ΔH_m_ (J/g)	X_c_ (%)
NEAT HDPE	142.88 ± 2.81	116.34 ± 2.81	150.81 ± 18.36	55.90 ± 1.19	138.81 ± 0.53	115.80 ± 0.18	193.89 ± 0.03	66.18 ± 0.01
NEAT HDPE5MA	137.01 ± 0.48	113.63 ± 1.40	187.03 ± 3.72	63.83 ± 1.27	137.20 ± 0.22	115.84 ± 0.84	193.63 ± 9.40	66.09 ± 3.55
HDPE10B	137.60 ± 1.25	114.85 ± 2.07	147.52 ± 3.33	55.94 ± 1.26	135.86 ± 0.18	117.46 ± 0.14	176.22 ± 2.19	66.84 ± 0.47
HDPE30B	137.73 ± 1.11	114.38 ± 0.07	117.96 ± 1.04	57.51 ± 0.51	136.12 ± 0.78	117.09 ± 0.06	136.31 ± 3.90	66.46 ± 1.90
HDPE10B5MA	137.04 ± 0.94	115.41 ± 3.25	140.57 ± 2.88	53.31 ± 1.09	138.26 ± 1.32	112.87 ± 0.36	173.24 ± 0.69	65.70 ± 0.26
HDPE30B5MA	136.54 ± 2.10	116.02 ± 2.79	109.17 ± 1.54	53.23 ± 0.75	136.47 ± 1.85	113.62 ± 0.02	138.35 ± 1.34	67.46 ± 0.35
HDPE10H	138.27 ± 0.59	116.08 ± 2.06	143.64 ± 3.97	54.47 ± 1.50	134.95 ± 0.40	117.47 ± 0.38	175.94 ± 6.76	66.72 ± 2.56
HDPE30H	133.80 ± 2.91	114.31 ± 0.58	107.83 ± 3.58	52.57 ± 1.75	134.32 ± 1.79	117.14 ± 0.23	118.20 ± 4.26	57.63 ± 2.08
HDPE10H5MA	137.57 ± 0.92	116.29 ± 1.99	149.97 ± 12.64	56.87 ± 4.79	136.24 ± 0.60	114.67 ± 0.13	171.17 ± 9.73	64.91 ± 3.69
HDPE30H5MA	134.65 ± 2.30	115.51 ± 0.87	100.81 ± 7.32	49.15 ± 3.57	133.90 ± 2.74	114.45 ± 0.59	128.82 ± 5.30	62.81 ± 2.59
HDPE10H20B	134.92 ± 0.11	117.46 ± 3.30	120.22 ± 6.54	58.62 ± 3.19	137.48 ± 3.93	117.60 ± 0.65	129.49 ± 0.44	63.13 ± 0.21
HDPE15H15B	137.10 ± 0.75	117.24 ± 2.74	112.70 ± 5.18	54.95 ± 2.52	134.31 ± 0.83	117.88 ± 0.02	127.51 ± 3.24	62.17 ± 1.29
HDPE20H10B	137.33 ± 1.61	117.54 ± 2.69	101.57 ± 0.14	38.52 ± 0.05	133.30 ± 0.57	117.75 ± 0.11	131.09 ± 4.63	63.91 ± 2.26
HDPE10H20B5MA	135.68 ± 0.47	117.49 ± 2.55	98.15 ± 0.72	47.85 ± 0.35	135.23 ± 0.01	117.80 ± 0.42	122.40 ± 0.31	59.68 ± 0.15
HDPE15H15B5MA	136.33 ± 1.22	117.54 ± 2.86	101.61 ± 0.23	49.54 ± 0.11	135.17 ± 1.39	115.72 ± 0.21	127.01 ± 5.90	61.92 ± 2.88
HDPE20H10B5MA	138.14 ± 3.68	117.93 ± 2.59	110.75 ± 12.99	54.02 ± 4.31	137.55 ± 1.72	114.69 ± 0.59	138.51 ± 8.85	67.53 ± 4.31
